# Built Environment Design and People with Autism Spectrum Disorder (ASD): A Scoping Review

**DOI:** 10.3390/ijerph18063203

**Published:** 2021-03-19

**Authors:** Giulia Tola, Valentina Talu, Tanja Congiu, Paul Bain, Jutta Lindert

**Affiliations:** 1 Department of Architecture, Design and Planning, University of Sassari, 07100 Sassari, Italy; giuliatola.20@gmail.com (G.T.); tancon@uniss.it (T.C.); 2 Countway Library of Medicine, Harvard Medical School, Cambridge, MA 02115, USA; paul_bain@hms.harvard.edu; 3 Department of Social Work and Health, University of Applied Science Emden/Leer, 26723 Emden, Germany; jutta.lindert@hs-emden-leer.de

**Keywords:** built environment, autism spectrum disorder, spatial requirements, scoping review

## Abstract

Built environment design can be considered as an influential factor in the quality of life of people with autism spectrum disorder (ASD). This scoping review provides an overview of the current available literature on the relationship between people with ASD and built environment in the specific field of the design of autism-friendly spaces. The literature review allowed the identification of three main factors to be considered when designing for people with ASD—the sensory quality, the intelligibility, and the predictability of the built environment—and, for each of them, a description of the spatial requirements that have been recognized as fundamental according to the specific spatial needs of people with ASD.

## 1. Introduction

Built environment design can be considered as an influential factor in the quality of life of people with autism spectrum disorder (ASD). In fact, the opportunity of people with ASD (and, more generally, of people functioning in an atypical way [1]) to autonomously interact with the built environment is often compromised (and sometimes denied) due to constraints arising from the mismatch between their specific spatial needs and the “built form” [2].

ASD is an umbrella term used for a range of heterogeneous neurodevelopmental conditions, characterized by: (i) difficulties in social communication and social interaction across multiple contexts and (ii) restricted and repetitive patterns of behavior, interests, and activities [3]. The ASD diagnosis is based on the observation of specific atypical behaviors within these areas.

The incidence of the disorder has increased significantly in recent years. The estimated global incidence is of 1 in 160 people [4]; a monitoring study carried out in eleven states (Arizona, Arkansas, Colorado, Georgia, Maryland, Minnesota, Missouri, New Jersey, North Carolina, Tennessee, and Wisconsin) of the United States in 2016 reported an incidence of 1 in 54 among 8-year-old children [5], showing an increase compared with the previous editions conducted in 2014 [6] and in 2012 [7] (incidence of 1 child in 59, and 1 child in 69, respectively).

The knowledge about autism has constantly evolved in the last fifty years: the definition of autism, as well as its causes and its diagnostic framing, have been and are being subjected to continuous integrations and remodeling [3,8,9,10]. The present definition of the disorder [3] includes autism within a spectrum or continuum of severity, emphasizing the need to consider the great variability of individual manifestations.

People with ASD tend to have a problematic relationship with the surrounding environment. Several studies have emphasized how the altered perception of sensory stimuli and processing of information coming from the environment is one of the main problematic issues in ASD [11,12,13]. The relevance of the different perception of environmental sensory stimuli was also underlined by the updates of DSM-5, which introduced the sensory difficulties among the behavioral patterns not covered in previous versions: “hyper- or hypo-reactivity to sensory input or unusual interest in sensory aspects of the environment (e.g., adverse response to specific sounds or textures, excessive smelling or touching of objects, visual fascination with lights or movement)” [3]. Furthermore, the direct testimonies of people with ASD demonstrate that it is necessary to consider the anomalous perception of sensory stimuli that come from the environment as one of the main problems of autism [14,15,16,17,18,19,20,21,22,23].

Another behavioral pattern characterizing the interaction between people with ASD and the environment is the “insistence on sameness, inflexible adherence to routines, or ritualized patterns of verbal or nonverbal behavior (e.g., extreme distress at small changes, difficulties with transitions, need to take same route every day)” [3]. Recent research underlined the relation between the difficulty of people with ASD to adapt or reschedule planned activities, according to circumstances and contexts, and a different executive functioning [13,24].

The area of executive functions involves a set of cognitive skills (such as working memory, inhibitory control, flexibility, planning, and attentional control) that, among other things, allow individuals to re-define their behavioral responses to unexpected events and to reschedule their agenda [25].

To date, the necessity to reconsider the living environments also on the basis of the specific needs of people who function in an atypical way [1] has gained increasing importance in the overall debate and also in the urban planning and urban design fields.

Therefore, it is essential to investigate how the built environment impacts people with ASD and how it can be re-designed and re-organized to promote their autonomy and to enhance their overall quality of life. Starting from these considerations, the present scoping review aims to investigate the relationship between the built environment and ASD in order to provide an overview of the currently available literature in the field of the design of autism-friendly contexts, and to identify and describe the recurrent autism-friendly spatial requirements proposed in order to design environments according to the specific spatial needs of people with ASD, aiming to enhance their possibility to access and to use the spaces of their daily life.

## 2. Methods

We conducted a scoping review according to the “Joanna Briggs Institute methodology for scoping review” specifically referring to the search framework proposed by Arksey and O’Malley (2005) [26,27,28]. Referring to these guidelines we carried out a three-stage methodological process to explore the relationship between the built environment and people with ASD.

The steps are the following: (i) the literature searching, (ii) the selection of studies, according to defined eligibility criteria (reported according to the Preferred Reporting Items for Systematic Reviews and Meta-Analyses (PRISMA) flowchart), and (iii) the extraction and synthesis of data (see Table S4 in Supplementary Materials and Table 3). They are described in detail below.

### 2.1. Literature Searching

The search strategy consisted in a set of keywords and MesH (Medical Subject Headings) terms strictly related to the research question (see Table 1), combined with boolean operators (see attachment A in Supplementary Materials).

The search terms have been applied in the following databases: PubMed, Scopus, PsycINFO and Web of Science.

The search strategy also included a hand-search stage of relevant research monographies and recent grey literature (according to eligibility criteria), as explained in the following paragraph.

### 2.2. Inclusion and Exclusion Criteria

Inclusion and exclusion criteria have been defined according to the research question (see Table 2). Not only peer-reviewed studies but also some other contributions (relevant research monographies and recent grey literature) were included through hand searching on the basis that they have been frequently quoted in the same selected peer-reviewed studies.

Other reviews, dissertations, conference proceedings, editorials, and comments were not included as well as studies afferent to other disciplinary fields whose outcomes are not strictly related to spatial design (as, for example, medical and clinical studies). In fact, some terms and keywords adopted recur also in other disciplines with different specific meanings; this occurs, for example, with the term “architecture” when adopted in computing or neuroscience.

Studies included referred to the general population of people with ASD, without any limitation in the age and in the severity within the spectrum.

We included studies reporting and describing as outcomes spatial requirements, guidelines, or criteria for the design of an autism-friendly built environment.

### 2.3. Studies Selection

**Figure 1:**
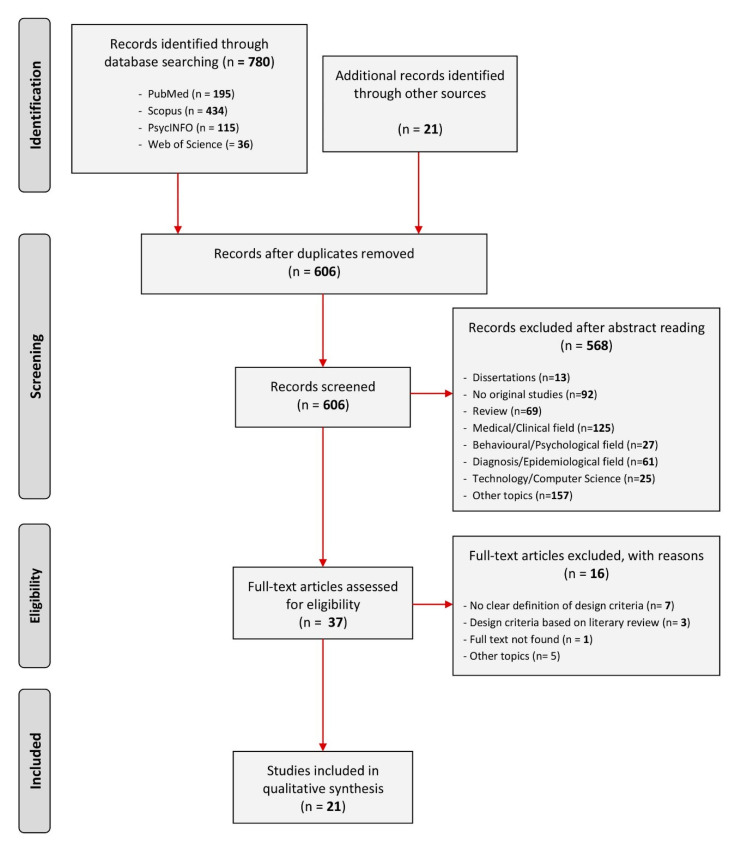
The studies selection process is reported according to the Preferred Reporting Items for Systematic Reviews and Meta-Analyses (PRISMA) flowchart model [29].

We screened the studies following two main stages: (i) checking through title and abstract and (ii) reading through the full text selected in the first stage. The screening process is reported according to the Preferred Reporting Items for Systematic Reviews and Meta-Analyses (PRISMA) flow diagram [29] (Figure 1).

In total, 801 studies were identified (*n* = 780 through database searching, and *n* = 21 through other sources). After the exclusion of 195 duplicates, 606 studies were screened through title and abstract checking. Full-text studies assessed for eligibility were 37, but at the end of the second stage a total of 21 met the inclusion criteria.

Even though they were related to the relationship between people with ASD and the built environment, the 16 studies excluded after the full text reading did not meet the inclusion criteria; *n* = 7 did not report clearly spatial requirements, *n* = 3 were based on review of other authors and designers, *n* = 1 the full-text was not found, and *n* = 5 focused on the topic from other different perspectives (e.g., test and description of assessment tools for educational spaces and residential facilities or navigation in virtual environment).

### 2.4. Data Extraction and Summary

We extracted and summarized relevant information through the full-text screening, referring to the Joanna Briggs Institute data charting [26,27] by using a data extraction grid developed by the authors (see Table S4 in Supplementary Materials and Table 3).

In the Table S4 (in Supplementary Materials) we included a first pointed summary of the identified design topics/guidelines. In the Table 3—Autism friendly spatial requirements—this pointed summary has been deepened.

Table S4 (in Supplementary Materials) reports, respectively, the following data:

1. main study characteristics (author/s, publication year, study design),
2. participants information (age range, gender, number of participants),
3. data collection method/s,
4. the built environment type (residential, learning, caring, outdoor spaces, sensory garden),
5. the study outcomes, and
6. the corresponding design topics/guidelines. The content of this last column is described in detail in Table 3.

## 3. Results

In total, 21 out of 801 studies identified met the inclusion criteria (Figure 1). This relatively limited number of selected pertinent studies proves that the topic of the relationship between people with ASD and the built environment in the specific field of the design of autism-friendly contexts need to be further explored.

All studies included have been published between 2008 and 2019, showing that this topic has gained recent attention.

Almost half of the studies have been developed in the USA (*n* = 9), 6 studies have been carried out in UK, and 5 in Egypt.

The authors of 15 out of 21 contributions are researchers or practitioners in architecture, landscape architecture, urban design, or urban planning; the remaining 6 studies have been carried out by scholars from different disciplinary fields (medicine, psychology, geography, etc.).

A well-defined set of design topics or guidelines has been identified in 19 out of 21 contributions, while we had to infer design recommendations from the remaining 2, since they did not develop them explicitly.

### 3.1. Study Design and Data Collection

The design of selected studies can be attribute to four approaches: case study design [12,30,31,32,33,34,35,36,37], co-design and participatory processes [38,39,40], surveys [41,42,43,44,45,46], and post-occupancy evaluation and intervention studies [47,48,49].

Most of the studies made use of interviews, questionnaires, and observation as methods of data collection. Precisely, 8 out of 21 studies conducted interviews [30,32,35,36,37,38,39,47], 8 out of 21 administered questionnaires [41,42,43,44,45,46,48,49], and a total of 6 carried out observations [32,36,38,47,48,49]. Only 2 studies also conducted focus groups [42,49]. Almost half of the studies did not adopt a single procedure but combined a set of previously cited methods and techniques [30,32,36,38,42,47,48,49].

Prevalent population of the studies included the network of professionals and ASD experts, parents and caregivers, and people with ASD (both adults and children around 13–20 years old).

### 3.2. Built Environment Type

Most of the studies focused on the definition of autism-friendly spatial topics or guidelines for learning environments devoted to childhood [12,35,37,39,40,42,43,44,46,47,48,49]. The characteristics of the residential environment have been investigated by 4 studies: three of them focused on adulthood [30,32,34] while the fourth one explored the adaptation of home spaces according to the needs of children [45]. Three studies deepened the relationship between the needs of people with ASD and the outdoor environment, specifically for the design of sensory gardens devoted to children [31,36] and to adults [38]. Two studies focused on the definition of spatial design guidelines for care environment [41] and for general autism-friendly indoor environments [33].

### 3.3. Study Outcomes and Design Topics/Guidelines

The last column of Table S4 (in Supplementary Materials) reports the outcomes and the topics or guidelines for the design of an autism-friendly built environment of the selected contributions.

For contributions identifying a well-defined set of design topics or guidelines [12,30,31,32,33,34,35,36,37,38,39,40,41,42,44,45,46,48,49], we opted to report the very same terms used by authors and not to alter the frameworks they proposed. We only omitted information not relevant to the research question, i.e., not concerning spatial issues.

We had to infer design recommendations from the contributions that did not develop a well-defined set of design topics or guidelines explicitly [43,47].

## 4. Discussion

Starting from the design topics or guidelines introduced by the authors of the selected contributions, we defined a set of spatial criteria for the design of an autism-friendly built environment.

These criteria were developed in such a manner that they could be applied to different scales, including the urban scale.

We divided spatial criteria in three groups on the basis of their relevance to specific features of the clinical descriptions of ASD: (i) sensory quality, (ii) intelligibility, (iii) orientation (Table 3 provides a detailed framework of the three groups of spatial criteria summarized below).

1. Spatial criteria pertaining to the necessity of improving the sensory quality of the built environment, and in particular to the necessity of reducing the impact of sensory stimuli (especially acoustic ones) coming from the environment. This group of spatial criteria refers to the DSM-5 diagnostic criteria “Hyper- or hypo-reactivity to sensory input or unusual interest in sensory aspects of the environment […]”.
  - Low arousal environment [12,30,31,32,33,34,35,36,37,38,39,40,41,42,43,44,45,46,47,48,49]: minimizing stimuli and details is one of the main requirements linked to the altered sensory processing in people with ASD. The screened studies focused particularly on visual, acoustic, and smell stimuli.
  - Transition spaces [30,34,36,37,38,39,40,42,43,44,48,49]: providing adequate transition between spaces in which people with ASD are exposed to different sensorial experiences is necessary to avoid sensory overload and to support the tasks of processing and integrating sensory information coming from the surrounding environment.
  - Quiet spaces [12,30,31,34,35,36,38,39,40,42,44,45,46,48,49]: providing spaces that allow for retreat is extremely useful for people with ASD to prevent or face sensory overload. Quiet spaces need to be designed following a well-defined set of spatial requirements in order to be comfortable and calming for people with ASD.
2. Spatial criteria pertaining to the necessity to make the built environment “intelligible”-and therefore actually accessible and usable-for people with ASD. To be intelligible, a built environment must have a simple spatial layout, facilitate orientation, and promote predictability. This group of spatial criteria refers to the DSM-5 diagnostic criteria “Insistence on sameness, inflexible adherence to routines, or ritualized patterns of verbal or nonverbal behavior […]”.
  - Clear and simple spatial layout [12,30,32,33,40,42]: providing a simple spatial organization helps people with ASD to navigate the space independently and with ease.
  - Visual relation [12,30,33,39,41]: guaranteeing the visual relationships among all components of the space and therefore giving the possibility of always having an overall view of the surroundings helps people with ASD to navigate the space with ease.
  - Predictability and routine [12,34,35,36,37,38,39,48]: providing a well-defined spatial structure contributes to enhance predictability and helps to avoid unexpected situations which may be problematic for people with ASD.
  - Circulation and possibility of choosing [30,31,37,39,40]: providing a hierarchy of spaces supports the possibility of choosing the type and level of social interaction and sensory stimulation.
  - Proportion and proxemics [12,33,34,44]: providing right proportions of spaces-both private and collective-helps to better perceive the mutual relationship between personal space and the environment.
3. Spatial criteria pertaining to the usefulness of using visual supports for helping people with ASD better navigate the environment. This group of spatial criteria refers to the DSM-5 diagnostic criteria “Insistence on sameness, inflexible adherence to routines, or ritualized patterns of verbal or nonverbal behavior […]”.
  - Visual supports [30,31,32,34,35,36,37,45,48]: using visual supports (with specific pictures, pictograms, colors, or small sentences) to report potentially critical situations and to indicate functions of different spaces helps people with ASD to interact appropriately with the environment.
  - Wayfinding [34,36,37,38,48,49]: using signs and wayfinding helps people with ASD to navigate the space independently and with ease.

These spatial criteria can be considered as autism-specific criteria. In addition to them, we should consider three criteria of general interest (see Table S5 in Supplementary Materials).

1. Identification of a quiet and accessible location [30,31,32,36,45]: choosing the neighborhood taking into account the physical and social dimension, the street network and mobility facilities, and the level of noise pollution is crucial to help people with ASD navigate the neighborhood and “using” available facilities and services.
2. Safety and security [12,30,31,32,33,34,36,37,40,41,44,45,49]: designing safe spaces is important to reduce risks, especially for those people with ASD who do not perceive danger.
3. Flexibility and customizing [12,31,35,36,39,41,46,49]: designing flexible spaces that can be configured for different functions allows to adapt surrounding environment to different needs of people with ASD.

The review allowed the identification of the main research outcomes about the topic of the relationship between people with ASD and the built environment. Starting from these outcomes, this work provided a systematization of spatial criteria for designing autism-friendly built environments.

This systematization can be used as a knowledge basis in autism-friendly design decision processes, including at the urban scale.

This work presents some limitations that we have already identified to suggest a future research agenda. We identified some main limitations.

First, we referred only to four databases: we acknowledge the possibility that some studies may have been missed for many reasons.

Second, we did not consider the great variability of individual manifestations of ASD. This great variability entails the difficult task of defining spatial criteria specifically defined to meet the spatial needs of each individual with ASD. Further research is therefore needed to define spatial criteria in such a way that they can be truly inclusive without being too generic.

The exclusion of some typologies of contributions such as conference proceedings, dissertations, and especially autobiographies was another limitation. As we have already highlighted, the topic of the design of autism-friendly cities and neighborhoods has gained recent attention and can be considered as an unexplored territory: therefore, it could be helpful to extend the review in order to include some selected relevant non-peer-reviewed studies, especially those written by authors with ASD.

Then, we argue that some experimental practices and policies in the field of the design of autism-friendly built environment should be considered as well, since they could give a worthwhile contribution to the advancement of research. Therefore, it could be useful to define a method for analyzing and monitoring the outcomes of these experimental practices and policies.

## 5. Conclusions

Studies in the field of the design of autism friendly built environment focused almost exclusively on closed and devoted spaces.

Most of them recommended a set of spatial requirements for designing autism-friendly learning spaces and, as a result, they considered the specific (spatial) needs of schoolchildren with ASD.

There are comparatively few contributions concerning the relation between the built environment and adults with ASD and they only focus on residential spaces.

No studies concentrated on the (re)design of the urban environment. In fact, the three existing contributions referring to this topic have been excluded because they do not meet the inclusion criteria: two are dissertations [50,51] and one refers to a research on urban navigation technologies based on virtual environment [52].

The present review is part of a broader research project on the relationship between people with ASD and the urban environment aimed at defining guidelines for designing an autism-friendly city. Although the identified spatial requirements have been defined referring to closed and devoted spaces, they are suitable for being usefully adjusted and then applied to the urban environment as well, in order to design an autism-friendly city.

## Figures and Tables

**Table 1:** Search settings.

Search Topics	Search Terms
Autism	autism spectrum disorder (MesH); autism; autistic
Built environment	environment design (MesH); residence characteristics (MesH); architecture (MesH); built environment; urban environment
Spatial design	design guide; design feature; sensory design; urban space; design criteria; neighbourhood

**Table 2:** Eligibility criteria.

Inclusion Criteria	Exclusion Criteria
The papers are peer-reviewed studies, monographies, and grey literature (reports and guidelines of case studies and realized projects).	Review articles, dissertations, conference proceedings, editorials, and comments.
The studies outcomes are design criteria, guidelines, spatial requirements to promote and design autism-friendly environment.	Studies afferent to other disciplinary fields whose outcomes are not strictly related to spatial design (as, for example, medical and clinical studies).
The outcomes are referred to the needs of people with ASD without any limitation in age and in severity within the spectrum.	

**Table 3:** Autism friendly spatial requirements.

DESIGN CRITERIA	SPATIAL REQUIREMENTS AND DESIGN RECCOMENDATIONS	REFERENCES
SENSORY QUALITY		
LOW AROUSAL ENVIRONMENT	VISUAL STIMULI Details: - reducing clutter by organizing the space with storage, shelves and cabinets; - minimizing visual stimuli and details eliminating nonessential visual materials; - avoiding to install too many openings on classroom wall, the outside view can be distracting. Lighting: - preferring natural lighting (avoiding direct sunlight) and ventilation; - avoiding fluorescent lighting because of the visual issue of flickering and the auditory issue of the low humming sound it emits, preferring LED lights instead; - using adjustable intensity lighting systems with diffuse light source to avoid glare; - providing plenty of shade, both with trees and shade structures as person with ASD are often photosensitive; - installing high-level windows to the exterior, to avoid visual distraction and limit glare effect. Materials and textures: - using a limited number of simple and non-reflective materials and textures; - preferring robust materials, with similar characteristics of domestic ones to avoid a too institutional and rigid environment; - using smooth and wide surfaces. Colours: - preferring soft, natural colours: whites, off-whites and pale pink tones are among the most popular choices (this cannot be generalized because it depends on the functioning level of the users and their sensory preferences/character); - limiting colour contrast; - using plants to separate environments devoted to different functions characterized also by different sensory stimulation level (e.g., use essences with soft colours along the paths and essences with bright colours only at the nodes). ACOUSTIC STIMULI Flooring: - using anti-trauma and sound-absorbing flooring (e.g., natural materials as softwoods like pine, natural sisal, or certain sound-absorbent vinyl, cork boarding); - using carpeting and wood furniture. Walls and Roofs: - adopting sound-reducing techniques for the external wall perimeter of the building, particularly if it is located in areas adjacent to high noise sources (heavy traffic streets, neighbouring public spaces like parks or schools); - avoiding high ceilings; - providing thick or soundproof walls by installing for example acoustical panelling along the lengths of the walls, colour coded with neutral tones arranged to follow a wayfinding scheme and without any sharp edging for mitigating echo. - providing build green roofs, whenever possible, to limit the impact acoustic of the rain. Background noises: - ensuring a good level of sound insulation between the different rooms; - installing higher efficiency fans with quieter performance with switches operated manually to avoid sudden activation; - reducing the openings in number as well as in size in areas requiring high acoustical quality such as a bedroom or speech therapy room; double or triple glazed windows and heavy curtains can also be used; - avoiding the “greenhouse” effect and providing a graduated series of acoustically modified rooms (depending on activities) to help children generalizing skills and not to become dependent upon an optimum acoustical quality. SMELL STIMULI Air quality: - ensuring a good ventilation for hyper-olfactory sensitive. Vegetation: avoiding smelly plants.	Ahrentzen, S., Steele K. (2009) [30] Barakat, H., Bakr, A., El-Sayad, Z. (2019) [31] Brand, A. (2010) [32] Deochand, N., Conway, A.A., Fuqua, R.W. (2015) [41] Gaines, K.S., Curry, Z., Shroyer, J., Amor, C., Lock, R.H. (2014) [42] Gaudion K., Mc Ginley, C. (2012) [38] Giofrè, F. (2010) [12] Humphreys, S. (2011) [33] Kanakri, S.M., Shepley, M., Varni, J.W., Tassinary, L.G. (2017) [43] Kinnealey, M., Pfeiffer, B., Miller, J., Roan, C., Shoener, R., Ellner, M. L. (2012) [47] McAllister, K., Maguire, B. (2012) [39] McAllister, K., Sloan, S. (2016) [40] Mostafa, M. (2018, 2014, 2010, 2008) [34,44,48,49] Nagib, W., Williams, A. (2018) [45] Piller, A., Pfeiffer, B. (2016) [35] Sachs, N., Vincenta, T. (2011) [36] Tufvesson, C., Tufvesson, J. (2009) [46] Vogel, C.L. (2008) [37]
TRANSITION SPACES	- Providing transition elements and areas between different activities/spaces to allows individuals to orient themselves or to rebalance sensory stimuli before experiencing environments with different functions and levels of sensory stimulation. - Locating sequence activities to introduce elements slowly. - Designing buffer areas such as gardens and outdoor learning spaces as transitional zone by allowing the sensory recalibration of the students while moving from a high stimulus function to a low-stimulus high focus activity.	Ahrentzen, S., Steele K. (2009) [30] Gaudion K., Mc Ginley, C. (2012) [38] Kanakri, S.M., Shepley, M., Varni, J.W., Tassinary, L.G. (2017) [43] McAllister, K., Maguire, B. (2012) [39] Mostafa, M. (2018, 2014, 2008) [44,48,49] Sachs, N., Vincenta, T. (2011) [36] Vogel, C.L. (2008) [37]
	- Grouping activities in accordance to sensory zoning rather than conventional functional zoning; this means localizing sensorial compatible functions together (e.g., “high-stimulus” functions like music, art, crafts and psychomotor therapy together while, on the other hand, “low-stimulus functions” as speech therapy, one to one instruction and general classrooms, requiring a high level of focus). - Dividing also the garden into different sensory areas on the basis on their stimulation level.	Gaudion K., Mc Ginley, C. (2012) [38] Kanakri, S.M., Shepley, M., Varni, J.W., Tassinary, L.G. (2017) [43] McAllister, K., Sloan, S. (2016) [40] Mostafa, M. (2014, 2008, 2010) [34,44,48]
	- Defining clear spatial distinction between the different activity/sensory stations by organizing a classroom or even an entire building into compartments (through furniture arrangement as bookcases, different flooring material as coloured tape or carpets squares, difference in level or even through variances in lighting). - Avoiding multi-functional and ambiguous areas to reduce sensory confusion.	Gaines, K.S., Curry, Z., Shroyer, J., Amor, C., Lock, R.H. (2014) [42] Mostafa, M. (2018, 2014, 2010, 2008) [34,44,48,49] Sachs, N., Vincenta, T. (2011) [36]
QUIET SPACES	- Providing calming and soothing areas for one-to-one teaching or interaction to retreat from overwhelming social situations; these spaces should be: - small and neutral in terms of sensory environment then with minimal distractions; - located by keeping the visual relationship with the surroundings, also to allow supervision; - partially and perceptually separated from the main space by using different design solutions: vegetation in outdoor spaces, with the location of bookcases in classrooms, with coloured masking tape marking off the area or through the placement of an area rug; - customizable to provide the necessary sensory input. - Positioning “difficult environment” as the dining hall close to the classroom dedicated to children with autism therefore they can get in first and are able to leave if are uncomfortable.	Ahrentzen, S., Steele K. (2009) [30] Barakat, H., Bakr, A., El-Sayad, Z. (2019) [31] Gaines, K.S., Curry, Z., Shroyer, J., Amor, C., Lock, R.H. (2014) [42] Gaudion K., Mc Ginley, C. (2012) [38] Giofrè, F. (2010) [12] McAllister, K., Maguire, B. (2012) [39] McAllister, K., Sloan, S. (2016) [40] Mostafa, M. (2018, 2014, 2010, 2008) [34,44,48,49] Nagib, W., Williams, A. (2018) [45] Piller, A., Pfeiffer, B. (2016) [35] Sachs, N., Vincenta, T. (2011) [36] Tufvesson, C., Tufvesson, J. (2009) [46]
CLEAR AND SIMPLE SPATIAL LAYOUT	- Designing a simple, well-defined space layout (e.g., providing a radial layout). - Preferring multifunctional circulation spaces to traditional corridors to allow the possibility of choosing the use of space.	Ahrentzen, S., Steele, K. (2009) [30] Brand, A. (2010) [32] Gaines, K.S., Curry, Z., Shroyer, J., Amor, C., Lock, R.H. (2014) [42] Giofrè, F. (2010) [12] Humphreys, S. (2011) [33] McAllister, K., Sloan, S. (2016) [40]
INTELLEGIBILITY		
VISUAL RELATION	- Ensuring a good visual relationship between different environments, interior and exterior of the building and also in the classroom to allow the teacher to see when a pupil is starting to get agitated or distressed. - Organizing the space to facilitate discreet supervision of the different environments. - Delimiting outdoor recreation areas.	Ahrentzen, S., Steele K. (2009) [30] Deochand, N., Conway, A.A., Fuqua, R.W. (2015) [41] Giofrè, F. (2010) [12] Humphreys, S. (2011) [33] McAllister, K., Maguire, B. (2012) [39]
PREDICTABILITY AND ROUTINE	- Emphasizing order, sequence and routine by organizing activities and functions according to ‘one-way’ circulation arrangement, referring to the daily schedule and routine, from inside the classroom to the building as a whole. - Making provision for positioning visual schedules for example with class timetable to help children be prepared for what it’s supposed to happen during the lesson, with visual aids to provide instructions for activities that can take place in different environments, by naming hallways and using color-coded zones, signs and numbering systems. - Preferring curvilinear walls: corners can hide dangers or unexpected things/situations and convey a sense of restlessness. - In outdoor environments include some elements of consistency (hedge, stone wall) to create predictable pattern.	Gaudion K., Mc Ginley, C. (2012) [38] Giofrè, F. (2010) [12] McAllister, K., Maguire, B. (2012) [39] Mostafa, M. (2010, 2008) Piller, A., Pfeiffer, B. (2016) [35] Sachs, N., Vincenta, T. (2011) [36] Vogel, C.L. (2008) [37]
CIRCULATION AND POSSIBILITY OF CHOOSING	- Providing opportunities for choice-making and for different levels of social interaction by including both personal spaces, to accommodate small groups (two or three people) and to enhance feelings of closeness, intimacy, and safety, and collective spaces for work and leisure. - Including additional area in the classroom to allow children with ASD having more personal space when they need; - providing a hierarchy of spaces, a centrally spacious circulation space giving the possibility to decide where to go and directly connected to the classroom. - Organizing the playground in different small areas by giving the possibility to children to choice for places to go and avoiding crowded situations; allowing direct access to this area from the classroom (specifically for the class dedicated to children with autism).	Ahrentzen, S., Steele K. (2009) [30] Barakat, H., Bakr, A., El-Sayad, Z. (2019) [31] McAllister, K., Sloan, S. (2016) [40] McAllister, K., Maguire, B. (2012) [39] Vogel, C.L. (2008) [37]
PROPORTION AND PROXEMICS	- Designing spaces with the right proportions, not too small or with too low ceilings that convey a sense of oppression but not even oversized and with too high ceilings. - Avoiding large open spaces or too long corridors (particularly when combined with nonsound absorbent finishing materials to not create echoes) that can turn into dead space for children who tend to orbit as a way of maintaining control of their bodies.	Giofrè, F. (2010) [12] Humphreys, S. (2011) [33] Mostafa, M. (2014, 2010) [34,44]
ORIENTATION		
VISUAL SUPPORTS	- Using circulation schemes reporting the visual daily schedules (often based on the “Picture Exchange Communication System”—PECS) helps students in transferring learning from the classroom (where these pictograms systems are used) to the hallways. - Adopting visual supports (images, words, colours) (i) to give information about the use and functions of different spaces, (ii) to give indications on how to use certain play and elements (e.g., potentially dangerous objects as household appliances, electrical outlets, windows, doors, etc.) and (iii) to report potentially critical points (e.g., stairs or slopes).	Ahrentzen, S., Steele K. (2009) [30] Barakat, H., Bakr, A., El-Sayad, Z. (2019) [31] Brand (2010) [32] Mostafa, M. (2010, 2008) [34,48] Nagib, W., Williams, A. (2018) [45] Piller, A., Pfeiffer, B. (2016) [35] Sachs, N., Vincenta, T. (2011) [36] Vogel, C.L. (2008) [37]
WAYFINDING	- Providing maps and creating evident paths by using colour coding and labels helps to support orientation. - In addition, enhancing visual features by introducing vegetation and colours (on flooring, walls, doors, etc.) makes easier recognisability and wayfinding of different activities, spaces and sensory areas.	Gaudion K., Mc Ginley, C. (2012) [38] Mostafa (2018, 2010, 2008) [34,48,49] Sachs, N., Vincenta, T. (2011) [36] Vogel, C.L. (2008) [37]

## Data Availability

See “2.1 Literature searching”.

## Supplementary Materials

The following are available online at https://www.mdpi.com/1660-4601/18/6/3203/s1, Attachment A: Search string, Table S4: Data of included studies, Table S5: Basic general spatial requirements.
