# Supplementary material for: Built Environment Design and People with Autism Spectrum Disorder (ASD): A Scoping Review
Source: Int J Environ Res Public Health. 2021 Mar 19;18(6):3203. doi: 10.3390/ijerph18063203 (PMC8003767; doi:10.3390/ijerph18063203)
Supplement: Supplementary file 1 [file ijerph-18-03203-s001.pdf]

Table 3. Data of included studies.

Author(s) (year)	Study country	Study design	Participants age range, gender	Admistration	Built environment type	Studies outcome	Design topics/Guidelines
Ahrentzen S. Steele K. (2009) [30]	USA	Case studies research	Staff and professionals of the buildings selected as cases study	On site visits; interviews	Residential environment for adults	Resident-based design goals	▪ Ensure Safety and Security<br>▪ Maximize Familiarity, Stability & Clarity<br>▪ Minimize Sensory Overload<br>▪ Allow Opportunities for Controlling Social Interaction & Privacy<br>▪ Provide adequate Choice & Independence<br>▪ Ensure durability<br>▪ Ensure Accessibility & Support in the Surrounding neighbourhood
Barakat, H., Bakr, A. El-Sayad, Z., (2019) [31]	Egypt	Empirical research	N.A.	N.A.	Outdoor environment for children	Design guidelines for therapeutic/ sensory garden	▪ Select a tranquil and quiet location<br>▪ Design for Security and Safe<br>▪ Design for Choice & Control and create a variety specialized spaces<br>▪ Design with Special Lighting Conditions in Mind<br>▪ Accommodate Needs for both Challenge and Rest<br>▪ Provide Calming Areas<br>▪ Child-nature Interaction<br>▪ Design for Ease of Maintenance<br>▪ Design for Future Spatial Flexibility<br>▪ Provide Visual Cues for Orientation
Brand, A. (2010) [32]	London, UK	People-centred design approach	Autism experts; family members of people with ASD; adult people with ASD	Interviews; observations on site; visits to supported living residences	Residential environment	Design themes for residential accommodations	Growth and Development:<br>▪ Independence<br>▪ Social Interaction<br>▪ Access<br>▪ Evolution<br>Triggers:<br>▪ Sensation<br>▪ Perception<br>▪ Refuge<br>▪ Empowerment

							Robustness:<br>▪ Safety<br>▪ Durability<br>▪ Ease of maintenance<br>▪ Tolerance<br>Support Tools:<br>▪ Communication<br>▪ Personal Support<br>▪ Unobtrusive Monitoring
Deochand, N., Conway, A.A., Fuqua, R.W. (2015) [41]	USA	Survey	Autism professionals (behaviour analysts and technicians, staff of treatment and residential centres); psychology professionals; psychology students	Questionnaire	Care environment	Beneficial design features for an intensive ASD treatment facility	▪ Health & Safety<br>▪ Damage Reduction<br>▪ Containment<br>▪ Observation<br>▪ Aesthetic
Gaines, K.S., Curry, Z., Shroyer, J., Amor, C., Lock, R.H., (2014) [42]	USA	Mixed-method approach:<br>1. Literary review<br>2. Pilot study<br>3. Survey research	Behavioural specialists; special needs teachers; general education teachers; ASD specialists; speech therapists; assistive technologists	Focus groups (n = 11); questionnaire (m = 604)	Learning environment	Environmental design factors to help reducing undesirable behaviours and contributing learning	▪ Space Organization<br>▪ Lighting<br>▪ Colour and Pattern
Gaudion, K., McGinley, C. (2012) [38]	Reading,UK	Co-design workshop	Autism experts; family members of people with ASD; adult people with ASD	Interviews; observations; visits on site for interacting with adult people with ASD	Outdoor environment for adults (sensory garden)	Design concepts to the redesign of the Kingwood College Garden (Reading, UK)	▪ Escape<br>▪ Sensory<br>▪ Transition<br>▪ Social
Assirelli, M.L. in Giofrè, F. (2010) [12] (Translation by the authors.)	Università di Roma La Sapienza, Italy	Case study	Facilities staff	Meetings	Social and health facilities	Design guidance for promoting comfortable and suitable places for people with ASD	▪ Spatial design<br>▪ Materials<br>▪ Acoustics<br>▪ Heating<br>▪ Lighting<br>▪ Ventilation
Humphreys, S., (2011) [33]	UK	Empirical study	N.A.	N.A.	Built Environment (not specified)	Design concepts for the development of projects for people with autism	▪ Calm, Order and Simplicity<br>▪ Proportion<br>▪ Minimal Detail and Materials<br>▪ Natural Light

							▪ Proxemics<br>▪ Observation<br>▪ Acoustics
Kanakri, S.M., Shepley M., Varni J.W., Tassinary L.G. (2017) [43]	Texas, USA	Survey	Special needs teachers	Questionnaire (May-September 2011)	Learning environment	Design features for acoustically friendly environments	▪ Layout<br>▪ Materials
Kinnealey, M., Pfeiffer, B., Miller, J., Roan, C., Shoener, R., Ellner, M. L., (2012) [47]	Toledo, USA	Multiple single-subject (6 weeks)<br>w 1–2: baseline (BL)<br>w 3–4: wall phase (WP)<br>w 5–6: lighting phase (LP)<br>WP: sounds-absorbing walls installation<br>LP: halogen lighting installation	Students (n = 4; age 13–20; male)	Behavioural responses observation; interviews (x3)	Learning environment	Behavioural response of acoustic and lighting environment modifications on children with ASD	▪ Acoustics<br>▪ Lighting
McAllister, K., Maguire, B., (2012) [39]	Northern Ireland, UK	Co-design approach (two years)	Teaching staff	Interviews	Learning environment	ASD-friendly classroom guidelines	▪ Threshold and entrance<br>▪ Cloakroom provision<br>▪ Sight lines entering the classroom<br>▪ Visual timetable<br>▪ High level glazing<br>▪ Volumetric expression<br>▪ Control<br>▪ Access to spaces<br>▪ Quiet room<br>▪ Floor area<br>▪ Storage
McAllister, K., Sloan, S., (2016) [40]	Northern Ireland, UK	Participatory design	Children with ASD (n = 17; age 13–18)	Workshop (4 sessions)	Learning environment	Design considerations for autism-friendly school environment	▪ Playground(s)<br>▪ Security<br>▪ Choice<br>▪ Noise and Comfort<br>▪ Circulation<br>▪ Legibility

Mostafa, M., (2008) [48]	Cairo, Egypt	1. Cross-sectional descriptive study;<br>2. Intervention study (control and study group, 1 academic year)	Teachers ($n = 25$); primary caregivers ($n = 58$)	1. Questionnaire<br>2. Direct observation of specific behavioural indicators to spatial design modification	Learning environment	1. Most influential architectural design elements on autistic behaviour ("sensory design matrix")<br>2. Guidelines to design environment for autistic users	▪ High enclosure and containment<br>▪ Low enclosure and openness<br>▪ Low ceilings and moderate proportions<br>▪ High ceilings and exaggerated proportions<br>▪ Use of intimate scale<br>▪ Use of open scale<br>▪ Orientation towards external views and elements of interest<br>▪ Use of activity focus to organize space<br>▪ Symmetrical organization<br>▪ Asymmetrical organization<br>▪ Use of visual or spatial rhythm<br>▪ Visually harmonious space with no contrast or discord<br>▪ Visually unharmonious space using accents and contrasts<br>▪ Use of dynamic and statically balanced spaces<br>▪ Use of unbalanced spaces<br>▪ Use of bright colours<br>▪ Use of neutral colours<br>▪ Use of warm colours<br>▪ Indirect natural lighting<br>▪ Direct natural lighting and views<br>▪ Noise and echo-proofing<br>▪ Use of smooth textures<br>▪ Use of rough textures<br>▪ Cross-ventilation<br>▪ Enclosed ventilation<br>▪ Organized compartmentalization using visual cues<br>▪ Spatial organization according to sensory characteristics<br>▪ Use of one-way circulation patterns to capitalize on routine

Mostafa, M., (2010) [34]	Cairo, Egypt	Case study design	N.A.	"Sensory Design Model" application to organize relationship between architectural environment and autistic needs	Residential environment	Design criteria for adapting housing to autistic use	Spatial quality:<br>▪ Acoustical Environment<br>▪ Tactile Environment: texture and closure<br>▪ Illumination<br>▪ Colour and Pattern<br>Spatial Organization:<br>▪ Sequencing and Routine<br>▪ Sensory Stimulus Zones<br>▪ Boundaries and Compartmentalization<br>▪ Furniture Distribution<br>▪ Navigation and Wayfinding<br>Spatial Orientation:<br>▪ Climatic Issues<br>▪ Natural Lighting, Visual Accessibility and iews<br>Spatial Integration: landscaping<br>▪ As a Sensory Curriculum<br>▪ Dynamics<br>▪ Gardening<br>Safety
Mostafa, M., (2014) [44]	Cairo, Egypt	Survey	Parents and primary caregivers (n = 100)	Ranking survey to identify prevalent sensory environment issue in order to apply sensory design matrix to school design	Learning environment	Development of spatial design criteria for the "Advance Center for Special Needs in Qattameya" according to the ASPECTSS™ Design	▪ Context and community<br>▪ Zoning<br>▪ Wayfinding, Navigation, Circulation and Spatial Sequencing<br>▪ Fire safety and evacuation<br>▪ Compartmentalization<br>▪ Escape spaces<br>▪ Sensory Zoning<br>▪ Transition Zones
Mostafa, M. (2018) [49]	Egypt	Post-Occupancy Evaluation of an existing pre-k-8th grade public charter purpose-built school for children with ASD	Teachers; school staff; parents; students	Questionnaire; behavioural observation in-class; focus groups	Learning environment	Design recommendations and interventions based on the application of the Autism ASPECTSS™ Design Index of an existing learning environments	▪ Acoustics<br>▪ Spatial Sequencing<br>▪ Escape Spaces<br>▪ Compartmentalization<br>▪ Transition Zones<br>▪ Sensory Zoning<br>▪ Safety

Nagib, W., Williams, A. (2018) [45]	USA	Survey	Parents of people with ASD ($n = 168$), random sampling;	Questionnaire	Residential environment for children	General design framework in developing friendly home environments	Physical environment:<br>▪ Sensory controlled environment<br>▪ Spaces for gross-motor equipment<br>▪ Freedom of movement<br>▪ Space for therapy<br>▪ Space for play<br>▪ Flexibility to accommodate future needs<br>Social environment:<br>▪ Proximity to the group but not in focus<br>▪ Space to retreat from overwhelming social situations<br>Symbolic environment:<br>▪ Promoting identity (personalization)<br>▪ Using visual symbols for activities and spaces
Piller, A., Pfeiffer, B. (2016) [35]	Philadelphia, USA	Qualitative descriptive approach (Sandelowski, 2000)	Preschool teachers; occupational therapists ($n = 13$)	Interviews	Learning environment	Sensory features that impact (in terms of support and inhibit) the participation in school tasks of preschool children with ASD	▪ Routines<br>▪ Modification and Sensory Support
Sachs, N., Vincenta, T. (2011) [36]	USA	Research-based design: examination of current available research, reference materials, literature, and personal interviews and observations	N.A	Interviews; observations	Outdoor environment for children	Design guidelines for outdoor environments for children with autism and special needs	▪ Select a location that is tranquil and quiet<br>▪ Include 5′-0″ minimum height fencing<br>▪ Provide smooth, wide pathways and surfaces<br>▪ Provide a clear edge along pathways<br>▪ Avoid specifying materials, including toxic plants<br>▪ Provide orientation maps<br>▪ Provide plenty of shade<br>▪ Provide transitions between spaces/activities<br>▪ Include some elements of consistency<br>▪ Sequence activities to introduce elements and ideas<br>▪ Provide fixed and non-fixed elements<br>▪ Provide plenty of visual aids and signage

							▪ Provide opportunities to overcame sensory issues<br>▪ Provide soothing areas<br>▪ Provide hammocks or hammock swings<br>▪ Build in challenge to help generalize
Tufvesson, C., Tufvesson, J. (2009) [46]	Sweden	Survey	Study one: School professionals and therapists (n = 125)<br>Study 2: Therapists, physiotherapists, psychologists, pedagogues, medical personnel, welfare officers; recreation instructors from ten Child- and Youth Habilitation Services (n = 137)	Questionnaires	Learning environment	Environmental factors affecting children with concentration difficulties (including people with ASD) in order to define guidance for design indoor school environments	▪ Space (Layout)<br>▪ View<br>▪ Interior furnishing<br>▪ Noise<br>▪ Light
Vogel, C., L., (2008) [37]	University of Wisconsin-Madison, USA	Research-based design: examination of current available research, reference materials, literature, personal interviews and observations	Parents; teachers; therapists; college students; adults with autism	Interviews	Learning environment	Design strategies for classrooms supporting children with ASD	▪ Flexible and Adaptable<br>▪ Non-Threatening<br>▪ Non-Distracting<br>▪ Predictable<br>▪ Controllable<br>▪ Sensory-Motor Attuned<br>▪ Safe<br>▪ Non-Institutional

Table 5. Basic general spatial requirements.

Design Criteria	Spatial Requirements and Design Reccomendations	References
Identification of a quiet and accessible home location	- Selecting a quiet neighbourhood and location with the least amount of distraction possible (noise from air conditioning compressors, adjacent traffic and high-pitched or humming noise can be overwhelming).<br>- Locating buildings in easily accessible areas provided by spaces and proximity services: supermarkets, health services, public spaces, green areas, etc.	Ahrentzen, S., Steele, K. (2009) [30]<br>Barakat, H., Bakr, A., El-Sayad, Z. (2019) [31]<br>Brand, A. (2010) [32]<br>Nagib, W., Williams, A. (2018) [45]<br>Sachs, N., Vincenta, T. (2011) [36]

Safety and security	- Choosing materials, structures, paving pattern and furnishings for ease of care and strength.<br>- Avoiding of sharp edges and corners and using soft surfaces to reduce the potential for injury.<br>- Preferring underfloor heating systems, radiant panels in false ceilings, frame radiators.<br>- Ensuring space visibility to ease supervision and transition by keeping transparency in windows and doorways.<br>- Delimiting outdoor play areas by using not obtrusive fencing.<br>- Avoiding toxic plants and using non-slip flooring.<br>- Installing raised windows, recessed shelving and lights.<br>- Ensuring adequate spaces ventilation (natural or forced).<br>- Locating staff room and principal's office centrally in the school, positioning junior classroom between the older children's classrooms.	Ahrentzen, S., Steele K. (2009) [30]<br>Barakat, H., Bakr, A., El-Sayad, Z. (2019) [31]<br>Brand, A. (2010) [32]<br>Deochand, N., Conway, A.A., Fuqua, R.W. (2015) [41]<br>Giofrè, F. (2010) [12]<br>Humphreys, S. (2011) [33]<br>McAllister, K., Maguire, B. (2012) [39]<br>McAllister, K., Sloan, S. (2016) [40]<br>Mostafa (2018, 2014, 2010) [49, 44, 34]<br>Nagib, W., Williams, A. (2018) [45]<br>Sachs, N., Vincenta, T. (2011) [36]<br>Vogel, C.L., (2008) [37]
Flexibility and customizing	- Designing a flexible space by allowing rearrangement and changes (e.g., to subdivide for group or individual work) without time-consuming renovations or costly by providing non-fixed elements.<br>- Giving the opportunities to customise and modify the living space.<br>- Considering modular templates to help sequence the classrooms more efficiently.	Barakat, H., Bakr, A., El-Sayad, Z. (2019) [31]<br>Deochand, N., Conway, A.A., Fuqua, R.W. (2015) [41]<br>Giofrè, F. (2010) [12]<br>McAllister, K., Maguire, B. (2012) [39]<br>Mostafa, M. (2018) [49]<br>Piller, A., Pfeiffer, B. (2016) [35]<br>Sachs, N., Vincenta, T. (2011) [36]<br>Tufvesson, C., Tufvesson, J. (2009) [46]
